# CO_2_ uptake on fruit wastes-derived activated hydrochars: systematic modeling of adsorption kinetics and isotherms

**DOI:** 10.1038/s41598-025-30726-7

**Published:** 2025-12-05

**Authors:** Sooraj Mohan, K. Ashwini, P. Dinesha

**Affiliations:** 1 Department of Mechanical and Industrial Engineering, Manipal Institute of Technology, Manipal Academy of Higher Education, Manipal, 576104 India; 2 Department of Artificial Intelligence and Machine Learning, Shri Madhwa Vadiraja Institute of Technology and Management, Bantakal, Udupi, 574 115 India

**Keywords:** Adsorption, Carbon capture, Char, Climate change mitigation, CO_2_, Kinetics, Chemistry, Environmental sciences, Materials science

## Abstract

Climate change mitigation requires efficient and low-cost approaches for carbon dioxide (CO_2_) capture, and valorization of fruit waste offers a sustainable pathway to address this challenge. This study establishes a systematic modeling framework for interpreting CO_2_ adsorption on activated hydrochars derived from banana and orange peels synthesized via hydrothermal carbonization. Multiple kinetic and isotherm models were evaluated using both the coefficient of determination ($\:{R}^{2}$) and the Akaike Information Criterion ($\:AIC$) to ensure robust comparison. Kinetic analyses revealed that the pseudo-second-order model ($\:{R}^{2}$ = 0.997, lowest $\:AIC$) and Elovich model best describe the uptake behavior, indicating chemisorption on heterogeneous surfaces. Equilibrium data were most consistent with the Tóth and Sips models ($\:{R}^{2}$ > 0.99), supporting monolayer adsorption coupled with micropore filling. By combining statistical rigor with mechanistic interpretation, this work advances understanding of the adsorption mechanisms of fruit waste-derived hydrochars and highlights their promise as scalable and sustainable sorbents for CO_2_ capture.

## Introduction

The increasing concentration of greenhouse gases, particularly carbon dioxide (CO_2_), in the Earth’s atmosphere has been identified as the primary driver of global climate change^1^. Anthropogenic activities such as fossil fuel combustion, industrial operations, and deforestation have significantly altered the natural carbon cycle, resulting in rising global temperatures, sea level rise, and frequent extreme weather events^2^. The Intergovernmental Panel on Climate Change (IPCC) Sixth Assessment Report underscores the urgency of immediate and sustained reductions in CO_2_ emissions to limit global warming to below 1.5 °C above pre-industrial levels^3^. Without substantial mitigation efforts, the anticipated consequences pose severe risks to ecosystems, public health, food security, and economic stability worldwide. In recognition of this global crisis, the United Nations adopted the Sustainable Development Goals (SDGs), among which SDG 13 specifically calls for urgent action to combat climate change and its impacts^4^. Achieving this goal necessitates a multifaceted strategy, involving technologies capable of actively removing CO_2_ from the atmosphere. Among the various carbon mitigation approaches, Carbon Capture and Storage (CCS) and Carbon Capture, Utilization, and Storage (CCUS) technologies have gained prominence as essential tools to complement emission reduction pathways, particularly in hard-to-abate sectors such as cement, steel, and chemical manufacturing.

Traditional capture techniques, such as chemical absorption using amine-based solvents, have demonstrated high capture efficiency; however, they suffer from drawbacks including high energy requirements for solvent regeneration, corrosion, and solvent degradation over time^5^. As a result, there is a growing interest in alternative low-energy, cost-effective, and environmentally benign materials for CO_2_ capture, particularly solid sorbents (^6–9^). The adsorbents, including activated carbons, zeolites, metal-organic frameworks (MOFs), and biomass-derived carbon materials, offer distinct advantages such as high surface area, thermal stability, and easier regeneration. Among these, activated chars synthesized from biomass sources^11^ have emerged as a promising class of sustainable adsorbents owing to their low-cost precursors, renewable origin, and the ability to tailor their textural and chemical properties via controlled synthesis methods.

Over the past two decades, extensive research efforts have been made to develop and evaluate materials for CO_2_ adsorption, with growing emphasis on low-cost, renewable, and scalable alternatives to conventional adsorbents. Many reports have been published in the literature concerning the CO_2_ capture using activated carbon and its kinetic and isotherm modelling (^12–14^). For example, Yang et al. investigated magnesite-based activated carbon that achieved a CO_2_ adsorption of about 1–3 mmol/g^15^. Similar CO_2_ uptakes were also reported with activated carbons synthesized from rubber seed shells^16,17^, okara powder waste^18^, etc. A slightly higher adsorption (3–6 mmol/g) was observed from the activated carbon produced from avocado seeds^19^, orange^20^ and banana peels^21^, commercial activated carbon(^10^), MIL-160(Al)^22^, etc. Regarding the kinetics and isotherm modelling, it is observed that pseudo-first order, pseudo-second order, and Avrami models are generally used by the researchers to depict the kinetics, while different isotherm models have been fitted to the experimental data. For example, Al-Absi et al. studied the CO_2_ capture on in-situ polymerized amines into PE-SBA-15 and obtained an uptake close to 1 mmol/g. The authors considered three kinetic models, pseudo-first order, pseudo-second order, and Avrami models, and found the pseudo-second order model to be the most conforming kinetics. Among the adsorption isotherms, the Langmuir dual-site model was the best fit to represent the kinetics. Similarly, other reports have used Sips^23^, Temkin^24^, Redlich-Peterson^25^, Dubinin-Ashtakhov^26^ isotherms, etc. to depict the adsorption behaviour.

From the review of the literature, a critical challenge in this domain is the inconsistent and often superficial application of adsorption kinetics and isotherm models. In many instances, researchers apply a limited subset of models, often selected arbitrarily, to fit the experimental data, without clear justification for their choice or a rigorous comparison of model performance. Similarly, while isotherm models such as Langmuir, Freundlich, and Temkin are frequently employed to describe equilibrium behavior, their relevance to surface heterogeneity, adsorption energy distributions, or multilayer formation is not always adequately discussed. In addition, the literature lacks a systematic methodology to distinguish between competing models based on both statistical robustness and physical plausibility. As a result, conclusions drawn from model fits may not accurately reflect the true adsorption mechanism, hindering the rational design of adsorbent materials.

In this context, the present study explores the CO_2_ adsorption capacity of activated hydrochars synthesized from fruit wastes, specifically banana and orange peels, under varying hydrothermal carbonization conditions. Unlike prior studies that rely on single kinetic or isotherm models, our approach systematically applies multiple models and evaluates them using both R^2^ and $\:AIC$. This ensures robust model selection and minimizes overfitting. By integrating statistical criteria with mechanistic interpretation, our framework provides a new methodological development for analyzing adsorption in fruit waste-derived hydrochars. By applying a structured model comparison framework, this work not only explicates the underlying adsorption mechanisms but also provides a methodology that can be effectively used for future experimental data. The findings highlight the chemisorptive nature and surface heterogeneity of CO_2_ adsorption on hydrochar materials and emphasize the value of using rigorous data interpretation tools to avoid misleading conclusions.

## Methods

### Activated hydrochar production

Locally available fruit waste from banana and orange peels were sourced. These wastes were oven-dried at 90 °C for 36 h until all the moisture was removed. Further, the dried peels were ground in a blender until the particles were smaller than 1 mm. The dried biomass was mixed with deionized (DI) water at a ratio of 1: 10 (w/v) and subjected to hydrothermal carbonization in a hydrothermal reactor at temperatures of 180 °C, 200 °C, and 220 °C. The obtained liquid was filtered, dried, and mixed with 6 M KOH solution, with an impregnation ratio (KOH: hydrochar) maintained at 3: 1 (w/w), stirred in a magnetic stirrer at 450 rpm for 3 h before neutralizing with HCl. After thorough cleaning with DI water, filtering, and drying, the KOH-exchanged hydrochar samples were subjected to activation at 700 °C in a vacuum furnace for 3 h. It should be noted that the KOH activation step at 700 °C is energy-intensive, which introduces an environmental trade-off. However, this is partly offset by using abundant, otherwise discarded fruit wastes as feedstock. The physical properties of these activated hydrochars obtained from orange and banana peels have been reported elsewhere^20,21^. The hydrochars obtained from orange peel and banana peel are abbreviated as OP-XXX and BP-XXX, where XXX denotes the hydrothermal process temperature in °C.

### CO_2_ uptake measurements

The activated chars obtained were subjected to CO_2_ adsorption in a Micromeritics 3FLEX 3500 gas sorption analyzer. The measurements were done for 1 bar at 25 °C. The pressure was taken up from 0.001 bar and tested the adsorption up to a relative pressure of 1 bar. The obtained data are plotted to understand the appropriate kinetic and the isotherm models. A comparison of both the OP and BP hydrochars at varying temperatures are made to assess the performance of CO_2_ capture.

### Kinetic models

The different kinetic models considered in the present work related to CO_2_ uptake are presented here^27,28^. All the kinetic models were tested and overlayed over the experimental data to determine the most conforming model.

#### Pseudo-first order (PFO)

The PFO model is generally suited for physisorption occurring on a uniform surface. The model works in such a way that the adsorption rate is proportional to the unoccupied hydrochar sites whose representation is shown in Eq. (1). Although the model is capable for detecting early stages of adsorption, it often underestimates the mode of chemisorption.

1 $$\:q\left(t\right)={q}_{e}\left(1-{e}^{-{k}_{1}t}\right)$$

Where, $\:q$ is the amount of CO_2_ adsorbed (mmol/g) in time $\:t$, $\:{q}_{e}$ is the equilibrium adsorption capacity (mmol/g), and $\:{k}_{1}$ – is the rate constant (min^− 1^).

#### Pseudo-second order (PSO)

Similar to PFO, PSO model is assumed to have the adsorption rate proportional to the square of the unoccupied hydrochar sites. It is usually found to be more accurate compared to PFO. The PSO model assumes that the rate controlling step occurs due to the covalent binding between the active surface and the CO_2_ gas. The adsorption of CO_2_, $\:q\left(t\right)$ is modeled as shown in Eq. (2).

2 $$\:q\left(t\right)=\frac{{k}_{2}{{q}_{e}}^{2}t}{1+{k}_{2}{q}_{e}t}$$

Where, $\:{k}_{2}$ is the rate constant of the PSO kinetic model (g/mmol-min).

#### Half-order model

The Half-order model, also called Half-Langmuir model has been developed as an amalgamation of first and zero order kinetics. A perfectly fitting Half-Langmuir kinetic model implies that the adsorption rate decreases as the number of active sites available for adsorption reduces. The half-Langmuir model with a half-order rate constant, $\:k$ is represented in Eq. (3).

3 $$\:q\left(t\right)=\frac{{q}_{e}\sqrt{kt}}{1+\sqrt{kt}}$$

#### Elovich model

Elovich model is more suited for surfaces having heterogenous characteristics. A well fitting Elovich model may indicate that the surfaces may have varying energy levels. The model assumes that the adsorption rate decreases exponentially as the amount of adsorbed substance increases. The adsorption, $\:q\left(t\right)$ in an Elovich model is shown in Eq. (4).

4 $$\:q\left(t\right)=\frac{1}{b}\text{ln}\left(1+\alpha\:\beta\:t\right)$$

Where, $\:\alpha\:$ is the initial adsorption rate (mmol/g-min) and $\:\beta\:$ is the desorption constant (g/mmol).

#### Avrami model

Avrami model was developed based on crystallization kinetics. It considers into account different mechanisms like surface diffusion and diffusion within pores. Equation (5) shows the Avrami model and the parameter $\:n$, called the Avrami exponent in the equation indicates if the mechanism is only surface adsorption ($\:n$ = 1) or if there are any diffusion-limited cooperative effects. $\:k$ is the Avrami constant

5 $$\:q\left(t\right)={q}_{e}\left(1-{e}^{{\left(-kt\right)}^{n}}\right)$$

#### Intraparticle diffusion (Weber-Moris) model

Weber and Moris developed a model called Intraparticle diffusion (IPD) based on Fick’s second law (shown in Eq. (6)), called the Intraparticle diffusion model. It considers the different stages of adsorption like boundary layer diffusion, proceeding to intraparticle diffusion, and the final equilibrium stage.

6 $$\:q\left(t\right)={k}_{id}\sqrt{t}+C$$

Here, $\:{k}_{id}$ is the Intraparticle diffusion rate constant and C concerns with the boundary layer thickness. The value of C being zero depicts a strong dominance of intraparticle diffusion. A larger value of C reflects a thicker boundary layer, implying that surface adsorption or external mass transfer plays a stronger role in controlling the adsorption process. The relatively high C values observed here therefore suggest that film diffusion and surface interactions significantly influence CO_2_ uptake, in addition to intra-particle diffusion.

### Isotherm models

There have been many isotherm models that are suitable for predicting the behaviour of CO_2_ adsorption on activated char/carbon^27,28^. Similar to the kinetic models, all the isotherm models are presented here that will be tested for fitting the experimental data.

#### Langmuir

Langmuir adsorption isotherm assumes that the hydrochar surface adsorbs the CO_2_ molecules having a mono layer shape. It assumes that there is no variation on the surface energy characteristics like potential and that there is no interaction between adsorbed molecules. The equilibrium adsorption is given in Eq. (7).

7 $$\:q\left(P\right)=\frac{{q}_{m}bP}{1+bP}$$

Here, $\:q\left(P\right)$ is the amount of adsorbed CO_2_ at pressure $\:P$, $\:{q}_{m}$ corresponds to the saturated adsorption and is interpreted as the theoretical monolayer coverage, and $\:b$ is the Langmuir constant related to affinity whose value presents insights on strong affinity to CO_2_.

#### Freundlich

Freundlich isotherm, unlike Langmuir assumes a multilayer adsorption on a heterogenous surface. The model also considers non-uniform distribution of heat. The equation for Freundlich isotherm is depicted in Eq. (8).

8 $$\:q\left(P\right)={K}_{F}\sqrt[n]{P}$$

$\:{K}_{F}$ is the Freundlich constant, and $\:n$ is a dimensionless heterogenous factor whose value less than unity indicates cemisorption and a value more than one indicates multilayer pysisorption.

#### Tóth

Tóth model as shown in Eq. (9) is an extended correction for Langmuir model.

9 $$\:q\left(P\right)=\frac{{q}_{m}bP}{\sqrt[t]{1+{\left(bP\right)}^{t}}}$$

Where, $\:b$ is the affinity constant. The equation reduces to Langmuir isotherm for value of $\:t$ = 1. Here $\:t$ is the heterogeneity parameter and is expected to be below unity indicating a heterogenous characteristic for CO_2_ capture.

#### Sips

Sips isotherm model is an amalgamation of both Langmuir and Freundlich isotherms. At low pressures, the adsorption appears to be Frundlich and levels off Langmuir at higher pressures. The isotherm equation is presented in Eq. (10).

10 $$\:q\left(P\right)=\frac{{q}_{m}{\left(bP\right)}^{n}}{1+{\left(bP\right)}^{n}}$$

Here, $\:b$ is the affinity constant and $\:n$ is the heterogeneity parameter. A value of $\:n$ less than unity indicates heterogenous characteristic and a value close to unity depicts Langmuir behaviour.

#### Temkin

Temkin isotherm model is non-linear (as shown in Eq. (11)) and it accounts for the adsorbate and adsorbent interaction.

11 $$\:q\left(P\right)=\frac{RT}{\phi\:}\text{ln}\left(AP\right)$$

Here, $\:A$ and $\:\phi\:$ are Temkin constants, $\:R$ is the gas constant, and $\:T$ is the absolute temperature. Temkin isotherm model is an intermediate between Langmuir and Freundlich isotherms which is suited for moderate surface heterogeneity. As the coverage of gas increases in the surface, the model assumes that the heat of adsorption decreases.

#### Redlich-Peterson

Redlich-Peterson model is another amalgamation of Langmuir and Freundlich isotherms which can applied to a wide range of pressures as shown in Eq. (12).

12 $$\:q\left(P\right)=\frac{{K}_{R}P}{1+{a}_{R}{P}^{{\beta\:}_{R}}}$$

Here, $\:{K}_{R}$ and $\:{a}_{R}$ are isotherm constants, and $\:{\beta\:}_{R}$ is the heterogeneity parameter. Its value less than unity indicates Freundlich behaviour (heterogenous) and a value closer to unity depicts Langmuir behaviour.

#### Dubinin-Astakhov

Dubinin-Ashtakhov (D-A) model is based on the Polanyi theory and can be used to describe microporous filling of CO_2_ gas inside the activated char. Hence, the model is mainly based on adsorption potential. The relation to adsorption capacity is given in Eq. (13).

13 $$\:q\left(P\right)={q}_{m}\text{exp}\left[-{\left(\frac{{\Lambda\:}}{E}\right)}^{n}\right]$$

Here, $\:E$ is the energy of adsorption, $\:n$ is the heterogeneity parameter (heterogenous if the value is very high), and $\:{\Lambda\:}$ is the adsorption potential given by Eq. (14).

14 $$\:{\Lambda\:}=RT\text{ln}\left(\frac{{P}_{0}}{P}\right)$$

where, $\:{P}_{0}$ and $\:P$ are saturated vapor pressure of CO_2_ and equilibrium pressure respectively. The D-A model reduces to R-P model when $\:n$ = 2 and the interpretation of adsorption energy can be obtained from the value of E whose value less than 8 kJ/mol depicts physisorption and a higher value mostly indicates chemisorption. Table 1 summarizes the different kinetic and isotherm models used in this work.

**Table 1:** Summary of the different kinetic and isotherm models used in this work.

Name of the model	Equation
Kinetic models	
Pseudo-first order	$\:q\left(t\right)={q}_{e}\left(1-{e}^{-{k}_{1}t}\right)$
Pseudo-second order	$\:q\left(t\right)=\frac{{k}_{2}{{q}_{e}}^{2}t}{1+{k}_{2}{q}_{e}t}$
Half-order	$\:q\left(t\right)=\frac{{q}_{e}\sqrt{kt}}{1+\sqrt{kt}}$
Elovich	$\:q\left(t\right)=\frac{1}{b}\text{ln}\left(1+\alpha\:\beta\:t\right)$
Avrami	$\:q\left(t\right)={q}_{e}\left(1-{e}^{{\left(-kt\right)}^{n}}\right)$
Intraparticle diffusion (Weber-Moris)	$\:q\left(t\right)={k}_{id}\sqrt{t}+C$
Isotherm models	
Langmuir	$\:q\left(P\right)=\frac{{q}_{m}bP}{1+bP}$
Freundlich	$\:q\left(P\right)={K}_{F}\sqrt[n]{P}$
Tóth	$\:q\left(P\right)=\frac{{q}_{m}bP}{\sqrt[t]{1+{\left(bP\right)}^{t}}}$
Sips	$\:q\left(P\right)=\frac{{q}_{m}{\left(bP\right)}^{n}}{1+{\left(bP\right)}^{n}}$
Temkin	$\:q\left(P\right)=\frac{RT}{\phi\:}\text{ln}\left(AP\right)$
Redlich-Peterson	$\:q\left(P\right)=\frac{{K}_{R}P}{1+{a}_{R}{P}^{{\beta\:}_{R}}}$
Dubinin-Astakhov	$\:q\left(P\right)={q}_{m}\text{exp}\left[-{\left(\frac{{\Lambda\:}}{E}\right)}^{n}\right]$

### CO_2_ uptake prediction

CO_2_ uptake prediction will be carried out based on one of the most conforming kinetic models. Scipy library will be used in python to optimize the most suitable equilibrium concentration and the model constants. This way, a suitable equation will be developed to predict the amount of adsorption with respect to the time. In continuation, in this work, times taken to achieve 50%, 75%, and 90% of the equilibrium adsorption has been calculated. We know that the rate of adsorption severely declines over time. In this work, two materials are considered, OP and BP which have been synthesized at three different hydrothermal temperatures. Hence the amount of time required to achieve 50% and 90% will give insights on the probable decisions that can be made while selecting the best hydrothermal process conditions and biomass suitable for CO_2_ uptake. The CO_2_ uptake was measured in triplicate, and the average of the same has been provided in the manuscript.

### Model evaluation

All the kinetic and isotherm models were developed in a python environment. The python libraries used for the modeling are *pandas* for data handling, *numpy* for numerical analysis, *matplotlib* for plotting graphs, and *scipy* for optimizing the $\:{q}_{e}$ and $\:k$ values of the kinetic models. The model evaluation criteria (goodness-of-fit) applied for this simulation are the coefficient of correlation ($\:{R}^{2}$) and Akaike Information Criterion ($\:AIC$). While $\:{R}^{2}$ depicts the closeness of the fitted values to the actual experimental data, $\:AIC$ uses the residual sum of squares ($\:RSS$). Similar to Karimi et al. (Karimi et al., 2018), who assessed model adequacy using $\:{R}^{2}$, Adjusted $\:{R}^{2}$, and analysis of variance testing, the present study also employed $\:{R}^{2}$ as a measure of variance explained. However, to provide a more robust basis for model selection, we additionally used the $\:AIC$, which penalizes model complexity. This allows for a more rigorous comparison of competing models, reducing the risk of overfitting and ensuring the chosen model has acceptable goodness-of-fit. The uniqueness of $\:AIC$ is that overfitting will be penalized and hence is sensitive to more number of parameters considered for the model. The Eqs. 15 and 16 represents $\:{R}^{2}$ and $\:AIC$ respectively.

15 $$\:{R}^{2}=1-\frac{RSS}{\sum\:{({q}_{exp}-{\stackrel{-}{q}}_{exp})}^{2}}$$

16 $$\:AIC=n\text{ln}\left(\frac{RSS}{n}\right)+2k$$

Where, $\:RSS$ is given by $\:\sum\:{({q}_{exp}-{q}_{mod})}^{2}$, $\:{q}_{exp}$ is the experimental adsorption, $\:{q}_{mod}$ is the adsorption obtained from the model at the same corresponding data point, $\:{\stackrel{-}{q}}_{exp}$ is the mean of the experimental data, $\:n$ is the number of data points ($\:n$ = 86 for this work), and $\:k$ is the number of model parameters. A good model will then be considered based on the correlation coefficient and the Akaike criterion. If more than one model satisfies both these criteria, then the physics on which the model was developed will also be considered for the selection.

## Results and discussion

### Adsorption experiments

**Fig. 1:**
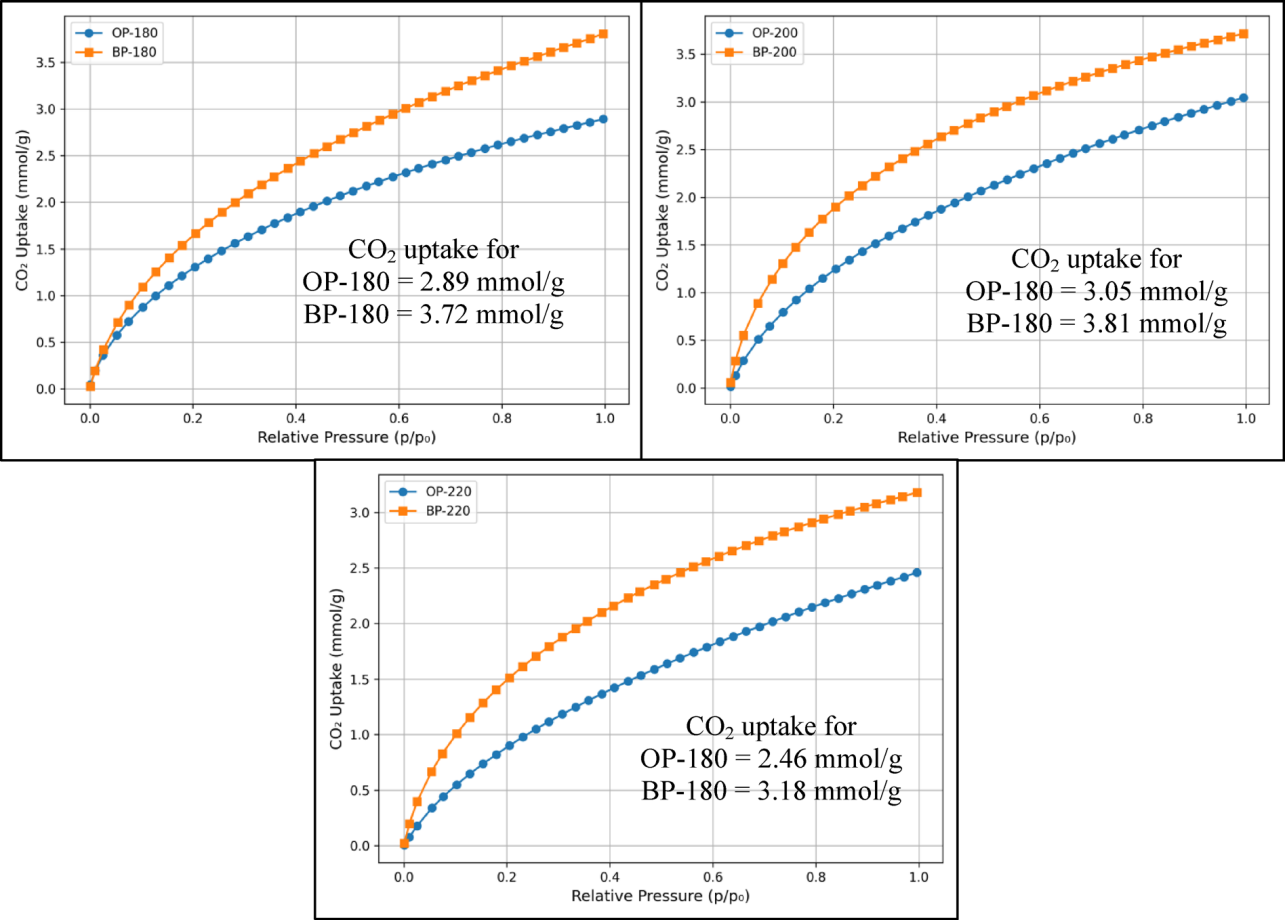
CO_2_ uptake isotherms of hydrochars obtained from banana and orange peels at (**a**) 180 °C, (**b**) 200 °C, and (**c**) 220 °C.

**Fig. 2:**
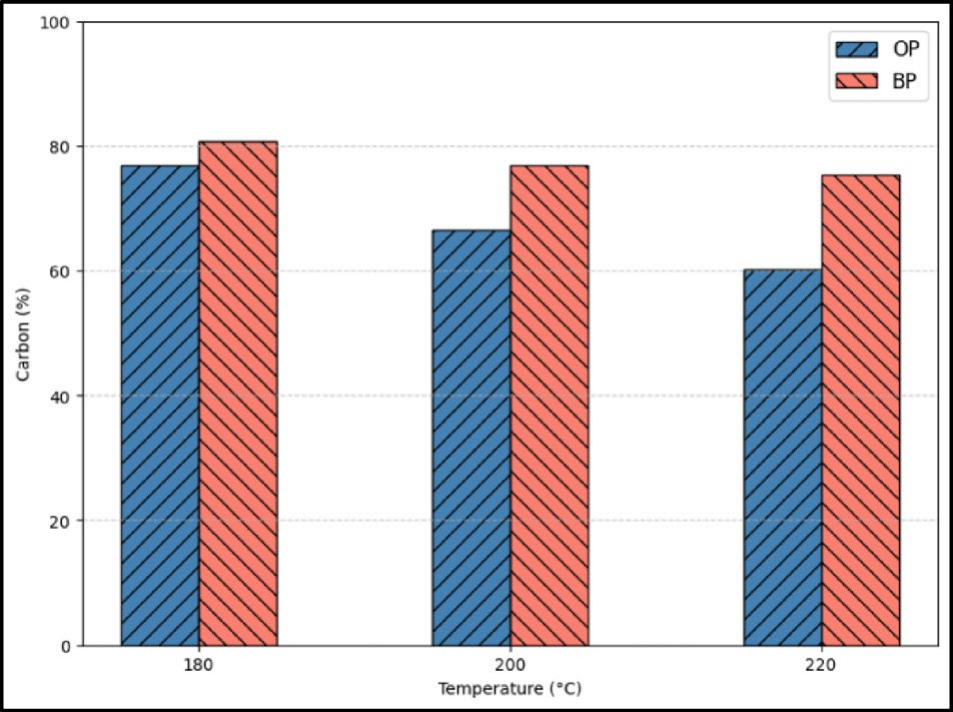
Elemental carbon composition (%) in OP and BP.

Three samples each of OP and BP, which were synthesized at three hydrothermal temperatures were considered in this study. The physicochemical properties of the hydrochars have been comprehensively reported in our previous works^20,21^. Briefly, BET surface areas were 154 m² g⁻¹ for BP-200 and 222 m² g⁻¹ for OP-200, with corresponding pore volumes of 0.08 and 0.158 cm³ g⁻¹. Both surface area and pore size distribution influence CO_2_ uptake. While a larger surface area provides more adsorption sites, micropores are especially effective at near-ambient pressures, as they create strong adsorption potentials for CO_2_. Mesopores mainly act as diffusion pathways and contribute less directly to capacity. Thus, abundant microporosity, together with an adequate surface area, is generally favorable for CO_2_ capture^29^. SEM images confirmed a hierarchical porous morphology, while FTIR spectra revealed abundant hydroxyl and carbonyl functional groups that are favorable for CO_2_ binding. These characteristics establish the structural basis for the adsorption performance discussed herein. Figure 1 (a)-(c) shows the amount of CO_2_ adsorbed at the three synthesis temperatures of 180, 200, and 220 °C. Among the three synthesis temperatures, hydrochars developed at 200 °C has a higher CO_2_ adsorption for both BP and OP hydrochars. The larger pore size created at 220 °C and lower surface area at 180 °C may have limited the CO_2_ uptake capacity of the activated hydrochars. A generic observation is that the activated hydrochar obtained from BP has a higher CO_2_ adsorption capacity for all the three synthesis temperatures. The higher adsorption of BP may have been mainly because of the higher elemental composition of carbon present in BP^21^ compared to OP^20^. Figure 2 shows the elemental carbon composition of OP and BP. Hydrochar obtained from banana peels has average composition at least 10% more carbon than the one obtained from OP. In the subsequent chapters, a systematic method will be followed to understand the kinetics and isotherm behaviours of both OP and BP hydrochars regarding its CO_2_ uptake.

It is shown in the literature that a higher carbon content indicates a greater degree of carbonization and structural ordering, typically accompanied by a lower concentration of oxygen-containing functional groups^30^. This results in a more hydrophobic and less polar surface, which enhances the physisorption of CO_2_ through dispersion forces and π–π interactions with the carbon basal planes^31^. Moreover, increased carbonization during KOH activation promotes micropore formation and pore widening, providing more accessible adsorption sites for CO_2_ molecules^32^. In our study, although OP-derived hydrochars showed higher BET surface area, the BP-derived samples exhibited higher carbon content and a more graphitized surface, which facilitated stronger CO_2_-carbon interactions and improved microporous structure. Therefore, the higher CO_2_ uptake observed for BP samples can be attributed to a combination of enhanced microporosity and greater carbonization, which together favor CO_2_ physisorption.

### Kinetic models

**Fig. 3:**
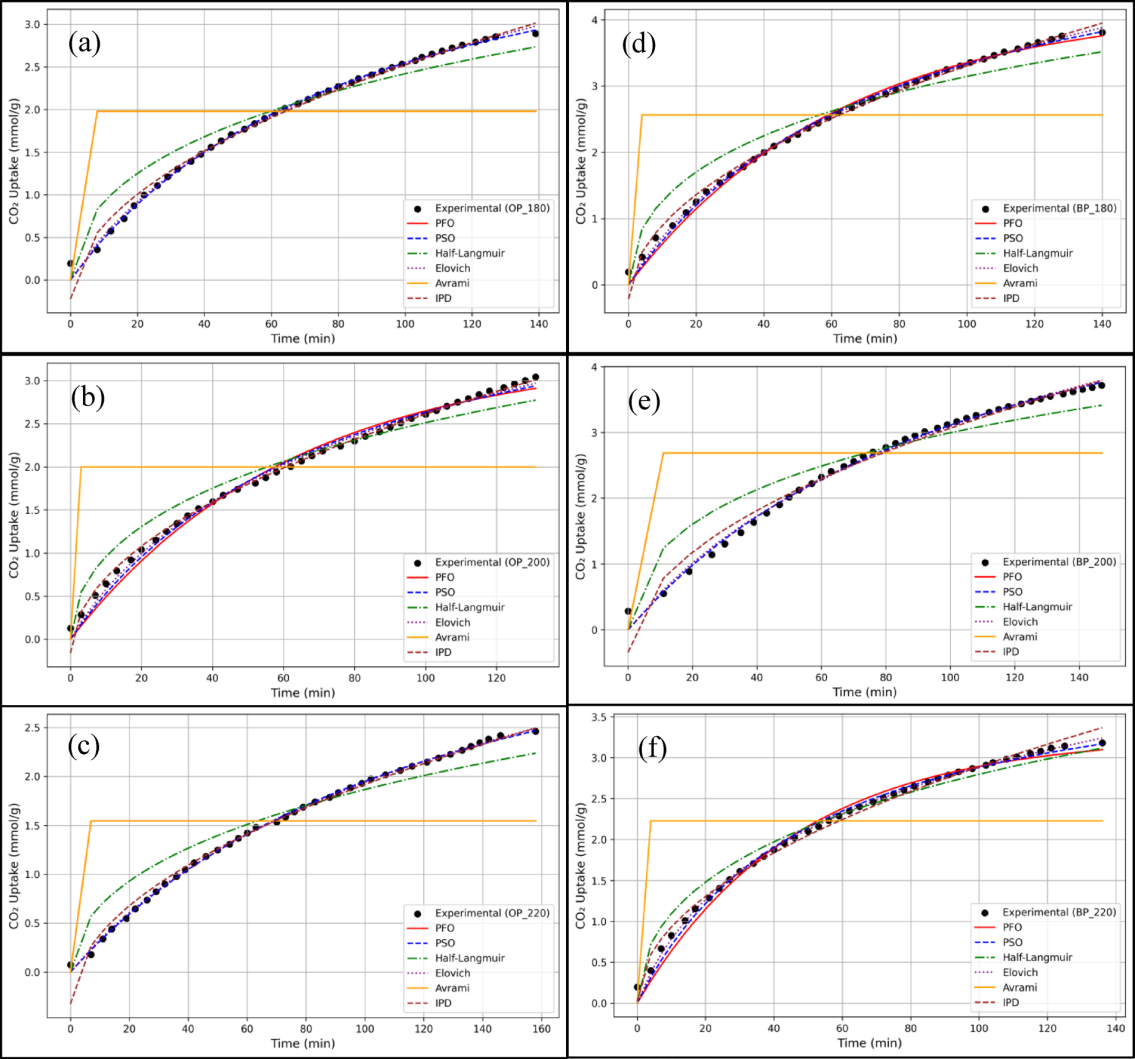
The different kinetic models fitted for (**a**) OP-180 (**b**) OP-200 (**c**) OP-220 (**d**) BP-180 (**e**) BP-200 (**f**) BP-220.

Figure 3 shows the kinetic models overlaid on the experimental CO_2_ uptake data. Table 2 shows the coefficient of determination for all the samples along with their AIC values. At the outset, for all the samples, it is clear that Avrami model does not fit the experimental data and hence does not capture the real essence of the kinetics. The mechanism that assumes nucleation and growth can also be eliminated from our understanding. This is important as it shows that the process may not involve anything complex, but rather a simpler mechanism like a pseudo-first or second order. Hence the surface may have a homogeneous or a nearly homogeneous characteristics. Since Avrami is a poor fitting model, the changes in the adsorbate and adsorbent structure may also be ruled out.

The second model to completely rule out is the pseudo-first order kinetics. Three of the six samples tested for returns with an $\:{R}^{2}$ of less than 0.2 and the average $\:{R}^{2}$ of 0.56 across all the samples. This inconsistencies in the fitting do not clearly capture the kinetics of the process. The mechanistic variability may be that some of the models may have superficially matched due to some coincidence and the inconsistency among 50% of the samples cannot be considered further for predictive analysis. A weak fit may be an indication of multi-step kinetics or presence of weak chemisorption mechanism.

Another model that does not effectively capture the kinetics is Half-Langmuir or Half-order model. This model returns with an $\:{R}^{2}$ of 0.93 indicating a relatively poor fit. Hence it can be inferred that the adsorption is not controlled by the early stage diffusion and hence is not the rate-limiting step. The results remove the assumption that the surface may be moderately homogeneous indicating that the physics is more of a pseudo-second order kinetics which assumes highly homogeneous surfaces. Although it could also be highly heterogeneous which can be captured by Elovich models. Hence a poor Half-Langmuir fit indicates that the process is probably faster and no transitional kinetics may be involved.

**Fig. 4:**
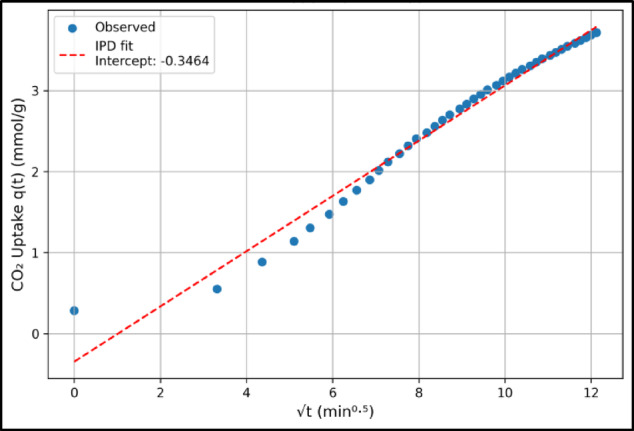
A plot of $\:q\left(t\right)$ against $\:\sqrt{t}$ for the IPD model in sample BP-200 indicating a y-intercept value of −0.346 mmol/g.

The other three kinetic models are closer fits to experimental data with an average $\:{R}^{2}$ value of 0.98 for IPD model and more than 0.99 for PSO and Elovich models. Three of the samples (OP-180, OP-220, and BP-200) had the highest $\:{R}^{2}$ and lowest $\:AIC$ for PSO model while the other three showed highest values of $\:{R}^{2}$ and lowest $\:AIC$ for the Elovich model, indicating that the IPD model had a minute lower representation compared to PSO and Elovich models. In addition, a plot of $\:q\left(t\right)$ against $\:\sqrt{t}$ for an IPD model must represent a straight line passing through the origin. Figure 4 shows an example plot for BP-200 which had an $\:{R}^{2}$ value of 0.975. However, the $\:q\left(t\right)$ against $\:\sqrt{t}$ plot clearly shows that the straight line is not a good fit with a y-intercept of −0.346 mmol/g. Similar plots were also obtained for the other samples with a significant y-intercept. These results reiterated that IPD was not the best kinetic model representing the CO_2_ uptake. Since the plots did not pass through the origin, it is evident that intra-particle diffusion was not the sole or rate-limiting step. Instead, film diffusion and surface chemisorption also contributed to the overall kinetics, which is consistent with the dominance of the PSO model. This indicates that CO_2_ uptake on these hydrochars is governed by a combination of surface-controlled and diffusion-controlled mechanisms rather than by intra-particle diffusion alone.

Concerning the remaining two models, high PSO fit may generally indicate that the mechanism is chemisorption, although it is also known that PSO may still fit a physisorption system. A good PSO fit essentially implies that the surface interactions are stronger than diffusion mechanisms. Since the hydrochar was treated with KOH, it may be considered as a chemically treated sorbent which is another characteristic of PSO model. Hence, a natural implication that chemisorption is the rate limiting step and that may occur between the hydrochar surface and the CO_2_ gas. It also can be inferred that the surfaces may have heterogeneity. A good Elovich fit, on the other hand, may depict different sites having varying energy levels. Since Elovich is a very good fit, it implies heterogeneous, disordered, porous surfaces and the rate of adsorption being nonlinear with time. The conformity with both the kinetic models inherently implies a chemisorption mechanism, and the degree of heterogeneous surfaces may have determined the compliance with either PSO or the Elovich models. Among the tested models, both the PSO and Elovich models described the kinetic data well. The strong fit of the Elovich model points to surface heterogeneity. However, the PSO model was ultimately favored because the calculated adsorption capacities closely matched the experimental values, providing stronger mechanistic validity. The Elovich model is more commonly applied to heterogeneous surfaces, which explains why it also provided a good fit in this case. The predominance of PSO therefore suggests that chemisorption at oxygenated surface functional groups (hydroxyl, carbonyl), as reported in our earlier works^20,21^, is the dominant rate-controlling mechanism.

Additionally, although OP-derived hydrochars exhibited higher BET surface area and total pore volume compared to BP-derived samples, the latter demonstrated superior CO_2_ uptake. This discrepancy highlights that adsorption performance is not solely dictated by overall surface area, but also by micropore proportion and the abundance of surface functional groups capable of strong CO_2_ interactions.

**Table 2:** Coefficient of determination ($\:{R}^{2}$) and $\:AIC$ criterion for determining the best fitting kinetic model.

Sample	Model	PFO	PSO	Half-Langmuir	Elovich	Avrami	IPD
OP-180	$\:{R}^{2}$	0.1418	0.9977	0.9285	0.9974	0.1418	0.9853
	$\:AIC$	−26.7	−263.0	−126.1	−258.1	−24.7	−189.5
OP-200	$\:{R}^{2}$	0.9879	0.9929	0.9498	0.9965	0.1316	0.9956
	$\:AIC$	−190.2	−211.7	−133.3	−239.4	−17.3	−230.7
OP-220	$\:{R}^{2}$	0.1177	0.9990	0.9152	0.9990	0.1177	0.9875
	$\:AIC$	−33.3	−303.0	−127.0	−302.7	−31.3	−203.7
BP-180	$\:{R}^{2}$	0.9942	0.9969	0.9259	0.9981	0.1417	0.9924
	$\:AIC$	−203.4	−228.4	−101.7	−248.4	−1.7	−192.5
BP-200	$\:{R}^{2}$	0.1676	0.9959	0.8845	0.9940	0.1676	0.9756
	$\:AIC$	−11.0	−223.5	−90.0	−208.6	−9.0	−152.3
BP-220	$\:{R}^{2}$	0.9888	0.9952	0.9713	0.9978	0.1537	0.9920
	$\:AIC$	−193.2	−226.7	−155.5	−258.5	−18.1	−206.4

### Isotherm models

**Fig. 5:**
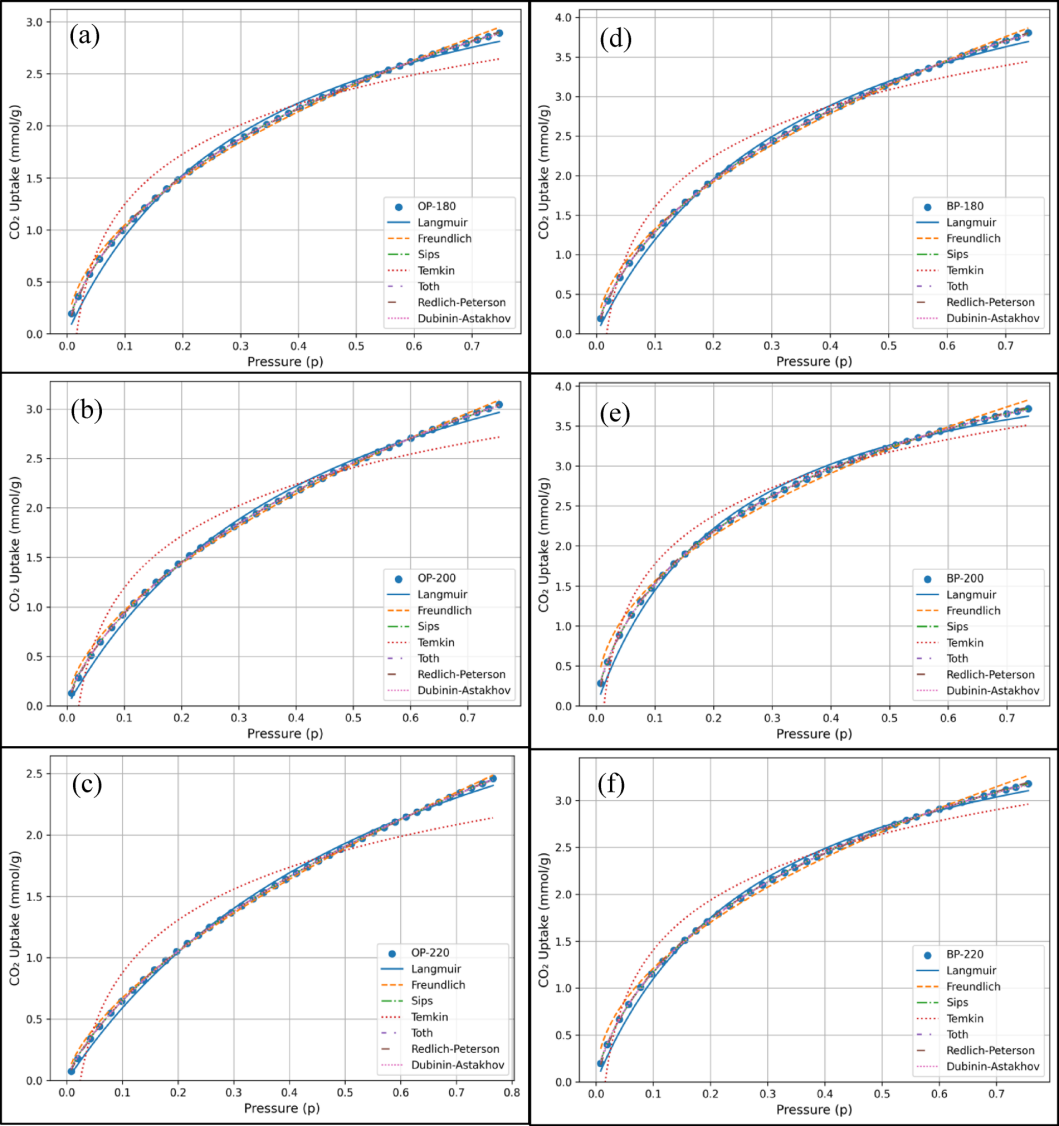
The different isotherms fitted for (**a**) OP-180 (**b**) OP-200 (**c**) OP-220 (**d**) BP-180 (**e**) BP-200 (**f**) BP-220.

The equilibrium adsorption data for CO₂ were evaluated using different isotherm models: Langmuir, Freundlich, Sips, Tóth, Redlich–Peterson, Dubinin–Astakhov, and Temkin which are presented in Fig. 5. The aim was to determine the most mechanistically and statistically appropriate model to describe the adsorption process. The correlation coefficients ($\:{R}^{2}$) and the $\:AIC$ for these models shown in Table 3. The Temkin isotherm, which assumes a linear decrease in adsorption energy with coverage, provided the poorest fit and was deemed unsuitable for this system. The Freundlich and Langmuir models, while returning acceptable fits, were however, inferior compared to the other isotherm models. Langmuir’s assumption of surface homogeneity and monolayer formation does not adequately describe the observed behavior which was also seen during the analysis of PFO kinetics. Freundlich, being entirely empirical, lacks saturation behavior and may have overpredicted the uptake at high pressure. The Dubinin–Astakhov isotherm, which is based on the potential theory of adsorption had a reasonably excellent fit that specifically addresses adsorption in microporous solids. It models the pore-filling mechanism rather than surface coverage and is particularly relevant for subcritical gases like CO_2_. The excellent fit indicates that pore-filling in microporous regions significantly contributes to the total adsorption capacity, which is expected in materials such as activated char. The Redlich-Peterson model, containing three adjustable parameters bridge the Langmuir and Freundlich models. However, unlike the Tóth and Sips models, the Redlich-Peterson equation is empirical and lacks direct physical meaning for its constants, making it less suitable for deriving mechanistic insight despite excellent statistical performance. The model is primarily a mathematical correlation developed to flexibly fit experimental adsorption data, rather than one derived from fundamental adsorption theory^33,34^. Unlike semi-theoretical models such as Langmuir or Dubinin-Astakhov, whose parameters correspond to physically interpretable quantities (like monolayer adsorption capacity, adsorption energy, or pore-filling potential), the constants in the Redlich-Peterson equation do not directly represent specific physical phenomena. They are fitting parameters that define the curvature and asymptotic limits of the isotherm, allowing the model to mimic both Langmuir and Freundlich behaviors depending on the value of $\:{\beta\:}_{R}$.

The Tóth isotherm, which yielded the highest $\:{R}^{2}$, modifies the Langmuir model by incorporating a heterogeneity parameter $\:t$. This parameter accounts for the energetic variation of surface sites, allowing the model to describe systems that deviate from ideal monolayer adsorption. The Tóth model effectively captures tailing behavior at high pressures, a common feature in CO₂ adsorption due to micropore saturation. The excellent fit and the physical relevance of its parameters indicate that the adsorption process is monolayer-dominated but occurs on an energetically non-uniform surface, typical of porous or functionalized adsorbents. These results are also supported from the discussion made in Sect. “Kinetic models”.

The Sips isotherm, which is another hybrid model combining the Langmuir and Freundlich equations, also showed excellent agreement with the experimental data. It behaves like the Freundlich model at low concentrations and transitions to Langmuir-type saturation at high concentrations. The Sips model’s ability to capture both initial heterogeneity and eventual site saturation confirms that CO_2_ adsorption in this system likely proceeds through a combination of heterogeneous binding at low pressure and monolayer formation at higher pressures.

Overall, the Tóth and Sips models are the most appropriate for describing CO_2_ adsorption in this system, with Tóth being slightly superior both statistically and mechanistically. Their performance indicates that the adsorption mechanism involves monolayer formation on a heterogeneous surface, coupled with significant micropore filling as indicated by the Dubinin-Astakhov model. The excellent fit of the Dubinin-Astakhov isotherm thus aligns with a micropore-filling mechanism, while the dominance of the PSO kinetic model further supports chemisorption contributions arising from these surface functionalities.

**Table 3:** Coefficient of determination ($\:{R}^{2}$) and $\:AIC$ criterion for determining the best fitting isotherm model.

Sample	Model	Langmuir	Freundlich	Sips	Temkin	Tóth	Redlich-Peterson	Dubinin-Astakhov
OP-180	$\:{R}^{2}$	0.9931	0.9976	1.0000	0.9292	1.0000	0.9999	0.9999
	$\:AIC$	−219.8	−262.6	−488.3	−126.5	−451.5	−388.5	−382.4
OP-200	$\:{R}^{2}$	0.9954	0.9984	0.9999	0.9111	1.0000	1.0000	0.9997
	$\:AIC$	−228.6	−270.1	−369.3	−110.4	−417.7	−461.6	−340.2
OP-220	$\:{R}^{2}$	0.9972	0.9990	0.9999	0.8904	1.0000	1.0000	0.9998
	$\:AIC$	−263.0	−303.1	−398.7	−116.7	−448.8	−488.8	−370.1
BP-180	$\:{R}^{2}$	0.9944	0.9976	0.9999	0.9227	1.0000	1.0000	0.9997
	$\:AIC$	−205.1	−239.5	−373.4	−100.0	−467.3	−461.8	−324.7
BP-200	$\:{R}^{2}$	0.9931	0.9936	1.0000	0.9543	1.0000	0.9998	0.9997
	$\:AIC$	−202.7	−205.8	−441.3	−127.1	−427.2	−350.9	−329.0
BP-220	$\:{R}^{2}$	0.9950	0.9948	1.0000	0.9446	1.0000	0.9999	0.9998
	$\:AIC$	−225.1	−224.0	−422.7	−129.1	−452.5	−384.6	−343.4

### Prediction of CO_2_ adsorption

**Fig. 6:**
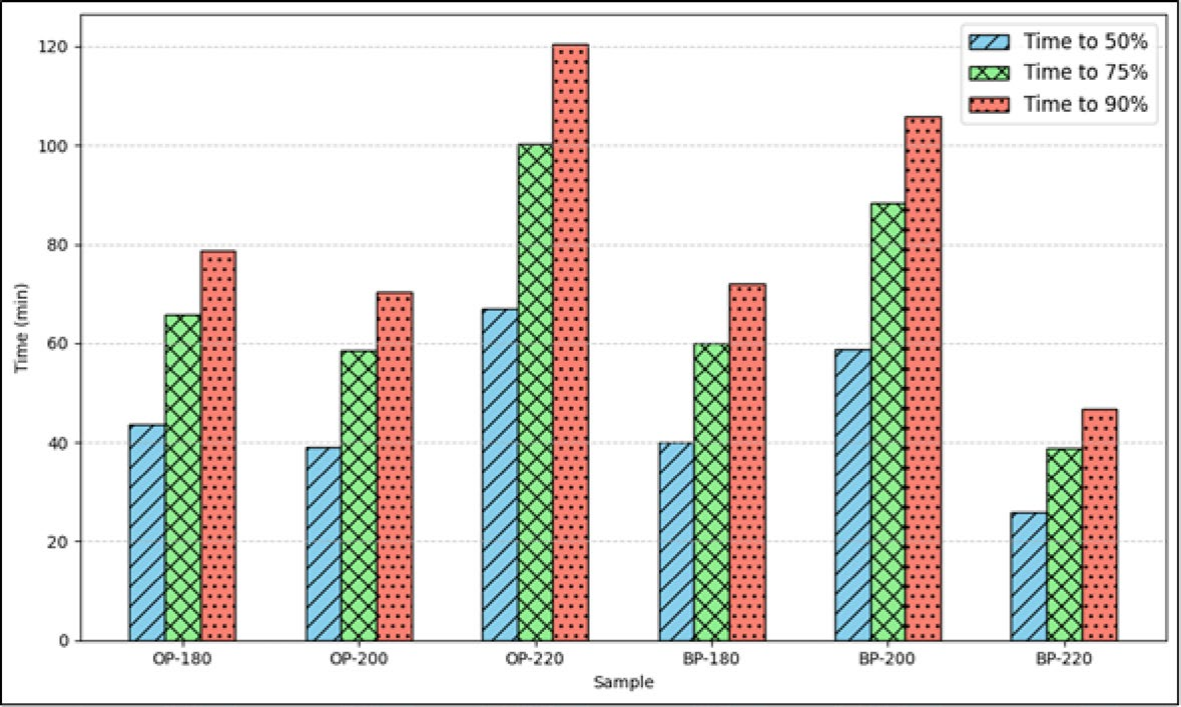
Time taken to reach 50%, 75%, and 90% of the equilibrium adsorption, $\:{q}_{e}$ for all the activated hydrochar samples using the PSO model.

It is seen from the kinetic studies that the PSO and Elovich model were equally fitting well to the experimental data. For further analysis, PSO model has been selected owing to the heterogeneity of the surfaces. The equilibrium adsorption ($\:{q}_{e}$) and the model constant, $\:{k}_{2}$ are now obtained from the PSO model. In the python environment, nonlinear least squares function (*curve_fit()*)is adopted using the *scipy.optimize* library. Table 4 shows the equilibrium values of $\:q$ and the corresponding value of $\:{k}_{2}$. These constants can now be used in Eq. (02) to predict the amount of CO_2_ adsorbed at any time $\:t$. However, in this work, a reverse analysis will be done wherein the amount of CO_2_ adsorbed will be fixed and the time taken to reach the specified amount is calculated. The amount of adsorption is fixed at a fraction of the final equilibrium adsorption, that are 50%, 75%, and 90% of $\:{q}_{e}$. The time taken to achieve these adsorption limits is calculated and plotted in Fig. 6 for all the samples.

**Table 4:** Values of $\:{q}_{e}$ and $\:{k}_{2}$ in the PSO model for different activated hydrochar samples.

Sample	$\:{\varvec{q}}_{\varvec{e}}$ (mmol/g)	$\:{\varvec{k}}_{2}$ (g/mmol-min)
OP-180	4.78	0.0024
OP-200	4.69	0.0027
OP-220	4.56	0.0016
BP-180	6.01	0.0021
BP-200	6.77	0.0013
BP-220	4.38	0.0044

Increased time indicates a relatively slower process of adsorption. Among the six samples, the average lowest time was obtained for BP-220 and the average longest time for OP-220. Considering the maximum amount of adsorption, all the OP samples faired poorly against the BP samples except BP-220. Both BP-180 and BP-200 showed a higher adsorption on the activated chars. BP-200 takes about 1.2 min to increase the CO_2_ adsorption by 1% of its equilibrium concentration. In comparison, BP-180 takes only 0.8 min for every 1% of the equilibrium concentration. Both the BP samples offer high rates of CO_2_ adsorption, but the hydrochar produced at 180 °C fairs relatively higher than its counterpart produced at 200 °C. These results call for an optimization study to be undertaken to assess the performance of CO_2_ adsorption by synthesizing hydrochars at temperatures between 180 and 200 °C. BP-220 also exhibited consistent behavior under the applied kinetic and isotherm models. The pseudo-second-order model provided the best fit, with the simulated equilibrium values closely matching the experimental uptake, similar to BP-180. However, the overall adsorption capacity of BP-220 was slightly lower than BP-180, which may be attributed to partial loss of surface functional groups at the higher activation temperature, despite the development of additional porosity. This trade-off between surface chemistry and porosity has been previously observed in fruit-waste-derived carbons.

### Practical implications and limitations

While the literature suggests MOFs and amine-functionalized carbons exhibit much higher CO_2_ capacities (> 4–6 mmol g⁻¹ at 1 bar), they suffer from high synthesis cost, regeneration instability, and scalability issues. In contrast, our fruit waste-derived hydrochars achieve moderate uptake (3.6 mmol g⁻¹), but their synthesis relies on abundant waste resources and low-temperature hydrothermal carbonization, offering clear advantages in sustainability and cost. This positions them as promising for large-scale, low-cost applications.

From a practical perspective, the hydrothermal carbonization of fruit wastes at 180–220 °C represents a relatively low-temperature and scalable route compared to conventional pyrolysis. The use of banana and orange peels as low-cost feedstocks provides an inherent economic advantage. However, this study did not investigate adsorbent regeneration or perform a detailed cost analysis. Future work will address cycling stability and benchmarking against conventional adsorbents (e.g., activated carbons, MOFs, amine-functionalized sorbents). These considerations highlight that while the present work advances kinetic and mechanistic understanding, further efforts are needed to translate hydrochars into viable CO_2_ capture technologies.

## Conclusion

This study systematically investigated the kinetic and isotherm modeling of CO_2_ adsorption on activated hydrochars derived from banana peel (BP) and orange peel (OP) via hydrothermal carbonization at 180, 200, and 220 °C. The results demonstrate that BP-derived hydrochars consistently outperformed OP-derived ones in CO_2_ uptake, which can be attributed to their higher elemental carbon content and more favorable pore structure. Kinetic analysis revealed that both the pseudo-second-order and Elovich models accurately described the adsorption behavior, jointly indicating a heterogeneous surface with chemisorption as the dominant mechanism. Among the isotherm models, the Tóth and Sips equations provided the best fits, suggesting monolayer adsorption coupled with micropore filling. Beyond model fitting, the study emphasizes that textural properties, particularly micropore abundance, play a decisive role in CO_2_ capture, and that the adsorption process is controlled by multiple steps involving film diffusion and chemisorption rather than intra-particle diffusion alone. The methodological framework applied here thus provides a robust approach to mechanistically evaluate CO_2_ adsorption on biomass-derived carbons.

Future work should address regeneration stability and reusability of these hydrochars, as well as comparative techno-economic evaluation against state-of-the-art adsorbents such as activated carbons, MOFs, and amine-functionalized materials. Further optimization of hydrothermal processing within the 180–200 °C range also appears promising for enhancing adsorption capacity. Further studies should also include a life cycle and techno-economic analysis to fully assess the CO_2_ mitigation potential of such adsorbents relative to their processing energy demands. By integrating systematic kinetic modeling with practical considerations, this study lays the groundwork for developing low-cost, sustainable sorbents from agricultural residues for CO_2_ capture.

## Data Availability

Data can be made available on a reasonable request.

## Abbreviations

$$\:A$$: Temkin constant
AIC: Akaike information criterion
$\:{a}_{R}$: Isotherm constant of Redlich-Peterson isotherm
$\:b$: Langmuir affinity constant
$\:C$: Intraparticle diffusion boundary layer thickness
CCS: Carbon capture and storage
CCUS: Carbon capture utilization and storage
CO_2_: Carbon dioxide
DI: Deionized
HTC: Hydrothermal carbonization
$\:{k}_{1}$: Rate constant in pseudo-first order
$\:{k}_{2}$: Rate constant in pseudo-second order
$$\:{k}_{F}$$: Freundlich constant
$\:{k}_{id}$: Intraparticle diffusion constant
KOH: Potassium hydroxide
$\:{k}_{R}$: Isotherm constant of Redlich-Peterson isotherm
MOF: Metal organic framework
$$\:n$$: Heterogeneity parameter
$$\:P$$: Pressure
P0: Saturated vapor pressure of CO_2_
PFO: Pseudo-first order
PSO: Pseudo-second order
$$\:q$$: CO_2_adsorption
$\:{q}_{e}$: Equilibrium concentration of CO_2_adsorbed
$\:{q}_{exp}$: Experimental adsorption of CO_2_
$\:{\stackrel{-}{q}}_{exp}$: Mean experimental adsorption of CO_2_
$\:{q}_{m}$: Saturated adsorption of CO_2_
$\:{q}_{mod}$: Kinetic model determined adsorption of CO_2_
$$\:R$$: Gas constant
$\:{R}^{2}$: Coefficient of correlation
$\:RSS$: Residual sum of squared
SDG: Sustainable development goal
t: Time

## Symbols

$\:\alpha$: Initial adsorption rate in Elovich isotherm
$\beta$: Desorption constant in Elovich isotherm
$\beta_{R}$: Heterogeneity parameter in Redlich-Peterson isotherm
$\wedge$: Adsorption potential in Dubinin-Ashtakhov isotherm
$$\phi$$: Temkin constant
